# Microquasar V4641 Sgr and Its Deep Investigation with LACT as a Super-PeVatron of Cosmic Rays

**DOI:** 10.34133/research.0872

**Published:** 2025-10-09

**Authors:** Zhen Cao, Dmitriy Khangulyan, Jiali Liu, Ruoyu Liu

**Affiliations:** ^1^ State Key Laboratory of Particle Astrophysics, Institute of High Energy Physics, Chinese Academy of Sciences, Beijing 100049, China.; ^2^ Tianfu Cosmic Ray Research Center, Chengdu 280134, China.; ^3^ University of Chinese Academy of sciences, Beijing, 100049, China.; ^4^ Astronomy Department, Nanjing University, Nanjing 210023a, China.

## Abstract

Several instruments, including LHAASO, have measured γ-ray emission from the direction of V4641 Sgr, a well known microquasar. The emission measured with LHAASO is best described as a power-law spectrum extending into the ultra-high-energy regime with no indication of a cutoff up to the energy of 0.8PeV. Thus, this source represents a perfect candidate of a super-PeVatron, which accelerates cosmic-ray particles beyond 10PeV. To prove this exciting hypothesis, a deeper investigation of this source is required. An important progress can be achieved using an array of Cherenkov telescopes, such as Large Array of imaging atmospheric Cherenkov Telescopes (LACT), observing the source at large zenith angles. In this paper, we show that already with the first 4 telescopes of LACT one will be able to constraint the key morphological features and the corresponding spectral variations in this source. This will provide the essential observational information for studying the acceleration of cosmic rays in this prominent source.

## Introduction

Origin of cosmic rays (CRs), which are a nonthermal ultra-relativistic component of space plasma, is one of the most fundamental problems in astrophysics. Many different types of sources can potentially contribute to this component, each creating a complex time-, space-, and energy-dependent distribution of CR particles. Furthermore, the CR spectrum contains contributions from different types of particles, such as electrons, protons, and heavier nuclei, and different CR sources may have varying capacities for accelerating these particles. Solving the problem of CR origin requires identifying the sources—so-called CR factories—that dominate specific parts of the CR spectrum.

Many experiments, both large-scale ground-based and space borne, aim to directly measure CR spectra in the vicinity of the Earth. These observations reveal the properties of the CR “soup”, which contains contributions from both nearby and distant sources. Determining the origin of CRs solely from these measurements presents a formidable challenge. Additional valuable information can be obtained by determining the CR spectrum in other locations, particularly near potential CR factories. Since in situ measurements of the CR spectrum are only possible within the solar system, the study of CR factories relies on astronomical observations. Identifying sources that produce ultra-high-energy (UHE; *E* > 100 TeV) γ rays is one of the primary goals of the Large High Altitude Air Shower Observatory (LHAASO).

The PeV range is a particularly significant part of the CR spectrum, as it exhibits a noticeable softening at around 3 PeV—the so-called knee. The conventional interpretation of this feature is that it marks the limit of efficient acceleration by typical Galactic sources, such as supernova remnants, or the onset of reduced CR confinement in Galactic magnetic fields. The presence of the knee implies that a substantial population of Galactic sources must be capable of accelerating particles to at least PeV energies. These sources are often referred to as PeVatrons. Furthermore, since the transition from Galactic to extragalactic CRs is expected to occur at higher energies—around the so-called ankle at ~5 EeV—this suggests the existence of Galactic super-PeVatrons, capable of accelerating particles well beyond 10 PeV [1–4].

One of the most promising approaches to identifying CR factories involves evaluating the maximum energy attainable by CR particles in source candidates. Such limits can be obtained using various approaches. In particular, the seminal paper by Hillas [5] derives the maximum energies under the assumption of a Fermi-type acceleration mechanism. In what follows, we derive a similar limit—identical in form to that in [5]—considering the electric potential drop across the source. Since the Lorentz force does not change a particle’s energy, energy gain occurs through the action of an electric field E. The maximum attainable energy for elementary particles is then limited by the potential drop:

$$E_{\max}≲eER\;,$$ (1)

where *R* is the source size.

In space plasma, the electric field is related to the magnetic field and plasma bulk speed: E=βB, where *β* is the speed in units of the speed of light. The total source luminosity *L* is related to the magnetic field strength in the outflow via a convenient parameter *σ*, dubbed magnetization, which is defined as the ratio of the Poynting flux to the source luminosity:

$$\frac{\beta cB^{2}}{4\pi }=\sigma \frac{L}{4\pi R^{2}}\;.$$ (2)

Comparing Eqs. (1) and (2) yields an illustrative limit:

$$E_{\max}≲\sqrt{\beta \sigma }\sqrt{\frac{e^{2}L}{c}}\;.$$ (3)

Adopting fiducial values of 0.1 for $\beta =0.1\beta_{-1}$ and $\sigma =0.1\sigma_{-1}$ (both strictly less than 1), we obtain the following constraint:

$$E_{\max}≲5\sqrt{\beta_{-1}\sigma_{-1}}\sqrt{\frac{L}{10^{39}\;\mathrm{erg}\;s^{-1}}}\;\mathrm{PeV}\;.$$ (4)

Equation (4) implies that the acceleration of PeV particles is possible only in mildly relativistic and magnetized outflows, with total luminosities exceeding $10^{39}\;\mathrm{erg}\;s^{-1}$. In the context of Galactic sources, such conditions naturally occur in microquasars. These compact binary systems consist of a stellar-mass black hole accreting matter from an optical companion and ejecting a mildly relativistic jet.

After contradictory early claims of detecting TeV and even PeV emission from microquasars, the current generation of γ-ray telescopes—such as HAWC, HESS, and LHAASO—has robustly established Galactic microquasars as a class of CR accelerators [6–9]. However, many crucial details, such as the composition of the accelerated spectrum and the cutoff energy, remain unresolved (see [10] and references therein). To deepen our understanding of the processes occurring in Galactic jets, further observations with instruments that provide better sensitivity and angular resolution in the UHE domain are required. If sufficient improvements are achieved, we will be able to constrain the maximum energy of the accelerated particles and localize the acceleration sites within these complex systems.

There are several approaches to measuring γ-ray emission with large-scale ground-based observatories: imaging atmospheric Cherenkov telescopes (IACTs; e.g., HESS, MAGIC, and VERITAS) and air shower arrays (ASAs; e.g., HAWC, Tibet ASγ, and LHAASO). Each approach has distinct advantages and limitations. ASAs provide the best sensitivity in the UHE domain at the cost of modest angular resolution. In contrast, IACTs offer superior angular resolution but suffer from declining sensitivity in the UHE range [11]. Therefore, a comprehensive study of microquasars requires a combination of both techniques, as demonstrated by the HAWC, HESS, and LHAASO observations of SS 433 [6–8]. In this letter, however, we show that a mid-size IACT array observing at high zenith angles can achieve both sufficient sensitivity and good angular resolution to constrain the acceleration process in a microquasar. As an exemplary case, we consider an in-depth observation of V4641 Sgr with the Large Array of imaging atmospheric Cherenkov Telescopes (LACT).

**Fig. 1.**
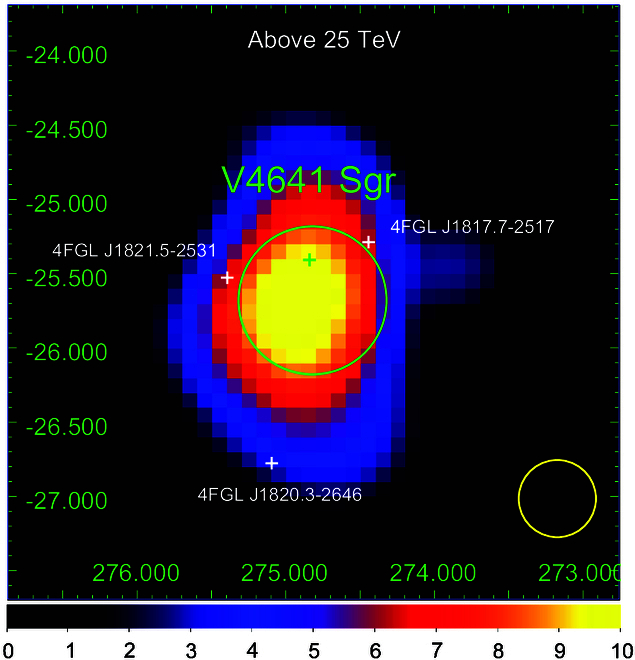
The morphology of V4641 Sgr in the high energy band (>25 TeV ) by LHAASO [8]. The green cross marks the position of the microquasar, and the green circle indicates its intrinsic extension. The yellow circle represents the width of the PSF that contains 39% flux from a point source.

Equatorial coordinates of V4641 Sgr are $\mathrm{RA}=274.84^{\circ }$ and $\mathrm{Dec}=-25.41^{\circ }$, so it can be seen from the LHAASO site (located at $29^{\circ }21^{\prime }27.6^{\prime \prime }\;N,100^{\circ }08^{\prime }19.6^{\prime \prime }E$) only at zenith angles larger than $55^{\circ }$. This limits the performance of LHAASO for this source as the daily exposure is only 3.3 h for the operational zenith angle range $≲60^{\circ }$. Still, KM2A of LHAASO has detected an extended source, LHAASO J1819-2541, spatially coincident with V4641 Sgr [8]. As shown in Fig. 1, the source shows an elongated morphology along the north–south direction, which can be described with a 2-dimensional elliptical Gaussian template with $\sigma_{1}=0.49^{\circ }\pm 0.08^{\circ }$ and $\sigma_{2}=0.1^{\circ }\pm 0.18^{\circ }$. The morphology can also be characterized with 2 point-like sources separated by $0.8^{\circ }$, although the goodness of fitting is slightly worse than the asymmetric Gaussian template. In the north–south direction, the source extends over an angular distance of $∼1.5^{\circ }$, which corresponds to 160pc at the distance of V4641 Sgr. The spectrum measured by LHAASO above 40 TeV can be described by a power-law function of a photon index of 2.84 with no significant indications on a cutoff in the spectrum. The highest energy of detected photons reaches 0.8 PeV, implying the source to be a super-PeVatron. The source was also detected by other γ-ray experiments. In particular, HAWC Collaboration reported a power-law spectrum with a photon index of 2.2 in the range of 18 to 217 TeV [9]. The fluxes measured with HAWC and LHAASO are consistent with each other within the overlapping energy range. Similarly to the findings of LHAASO, the morphology of the source measured by HAWC is consistent with either a 2 point-like sources or an asymmetric Gaussian template. The uncertain morphology of the source hinders discrimination between different physical scenarios, thus complicating understanding the details of the physical processes taking place in the source. It is still unclear if the emission detected with HAWC and LHAASO originates from the extended jets, or the 2 compact radiation zones caused by termination shocks of 2 jets, or a halo-like structure created by CR particles anisotropically diffusing away from the source. Observations by instruments with better angular resolutions will greatly facilitate our understanding of the nature of the source.

## Results

At the LACT site, V4641 Sgr rises to 35° above the horizon and starts to descend. If the observation starts when V4641 Sgr rises to 15° in elevation, the total exposure time will be 280 h in the observation season starting from October 2026. Hinted by LHAASO and HAWC observations, we assume the spectral energy distribution (SED) of the V4641 Sgr to be a power-law with an index of −2 with an exponential cutoff. We simulate the observational results of V4641 Sgr by P-1 for 3 scenarios of $E_{\mathrm{cut}}=0.3,0.5,1.1$ PeV. A simulation toolkit for the air shower development and the detailed detector responses [12] are used in the investigation.

**Fig. 2.**
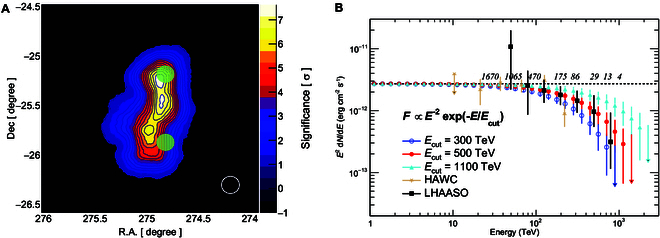
Synthetic significance map and SED of V4641 Sgr expected in 1 year after starting observations with P-1 telescope array. (A) The color map and contours show the expected significance of the detection of V4641 Sgr in energy range from 30 to 100 TeV, assuming the morphology suggested by HESS [14]. The expectation of 2 individual point-like sources hinted by HAWC observation [9] is also represented by 2 green spots, which indicate the significance of 14 *σ*. (B) Expected SEDs in scenarios with assumptions of the cutoff energies at 300, 500, and 1,100 TeV. The expected events with energies greater than 30, 50, 100, 200, 300, 500, 700 and 1,000 TeV are shown in the figure in case of *E*_cut_ = 0.5 PeV. The spectrum measured by LHAASO (black solid squares) [8] and HAWC (orange triangles) [9] is also presented here.

In Fig. 2A, the detailed morphology of the source between 30 and 100 TeV is shown in the significance map of the source region. Two scenarios are displayed in the same panel. One includes 2 individual point-like sources, represented by the 2 hot spots in green, as hinted by HAWC’s previous observation [9]. The highest significance reaches 14 *σ*. The other is a more complex elongated structure, shown with the color map and contours, favored by the HESS preliminary results [13] at lower energies below 10 TeV. With the point spread function (PSF) indicated by the white circle in the bottom right corner of Fig. 2A, we will be able to distinguish these 2 scenarios in the first operation season. Regarding the spectral measurement, as shown in Fig. 2B, our observation will be able to differentiate between the cutoff energies *E*_cut_ with sufficient statistics. For example, the number of collected γ rays above 100 TeV will be greater than 370 even in the case of $E_{\mathrm{cut}}=0.3$ PeV; however, no event is expected above 1 PeV. The statistical uncertainty in photon flux is still less than 10% at 100 TeV. On the other hand, if *E*_cut_ was close to 1.1 PeV, more than 16 events will be expected above 1 PeV. The deviations between the 2 scenarios and the model with *E*_cut_ = 0.5 PeV could be as large as 7.1 *σ* and 5.7 *σ*, respectively, estimated by using γ rays above 100 TeV.

Furthermore, if there is a hybrid contribution of the source from particles of different origins, we may potentially resolve different components of the source (e.g., a component related to jet and the other related to the central black hole). According to our simulation, ~1,200 γ-ray events were expected between 30 and 100 TeV for the assumed spectrum, which is sufficient for the proposed morphological analysis. Even if the source is contributed by a single particle component (the jet or the central black hole), we may measure the spatial variation of the spectrum at different regions. Energy-dependent morphology would be another important property of the source. We may perform the morphology analyses in 3 energy bins separately, for instance, in 3 to 30 TeV, 30 to 100 TeV, and >100 TeV, with high statistics. These analyses can provide important clues to the particle injection and transport within the source, and help us to locate the accelerator and uncover the nature of the source.

## Conclusion

LACT is a next-generation array of Cherenkov telescopes currently under construction at the LHAASO site. The first 4 telescopes (P-1) in the array are expected to deliver excellent performance, particularly for the observation of γ-ray sources at large zenith angles.

Among all interesting targets for LACT, the microquasar V4641 Sgr is the most promising one, due to both the importance of the underlying physics and the potential for significant improvement over current observational results. During the observation season from October 2026 to April 2027, we anticipate a precise spectral measurement that can distinguish between different spectral cutoff energies—an essential diagnostic for determining the maximum particle acceleration energy achievable by the source.

Moreover, the investigation suggests that LACT’s exceptional angular resolution, combined with high-statistics measurements, will enable a detailed reconstruction of the source morphology. This will provide critical insights into the radiation scenarios, especially for photon emission near 1 PeV.

These measurements are expected to yield crucial information about one of the most powerful in the Galaxy candidates for CR accelerator operating at energies well above the “knee”, with results expected in the near future.

LACT is a next-generation array of Cherenkov telescopes under construction on LHAASO site. The first 4 telescopes (P-1) built in this array promise excellent performance in the observation of γ-ray sources at large zenith angles. The microquasar V4641 Sgr is one of the best targets for LACT in terms of importance of the underlining physics and significance of the expected improvements in respect to the current state. In the observation season from October 2026 to April 2027, we expect a precise spectral measurement that helps to differentiate the cutoff energies of the spectra, which indicate the maximal energy of the particle acceleration capability of the source. The investigation also shows that the morphological structure of the source can be measured with high precision, thus revealing the crucial structures associated with the radiation mechanism, particularly for emission of photons around 1 PeV, due to the excellent angular resolution of LACT and high statistical measurement. Crucial messages about the most powerful potential Galactic source of CRs at energy well above the “knee” will be available in notably short future.

## Methods

**Fig. 3.**
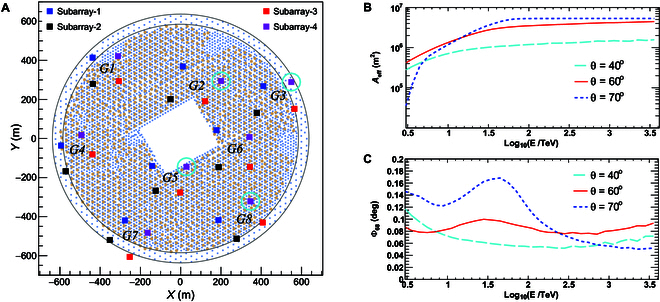
The layout of LACT, its P-1, and the performance. In (A), squares represent telescopes. Four colors indicate 4 configurations of combinations of the telescopes in 4 arrays that can be operated to observe 4 sources simultaneously. Basic configuration is 8 groups of 4 telescopes. Spacing between telescopes and groups is ~120 m and ~420 m, respectively. The 4 telescopes highlighted by cyan cycles form the sub-array of LACT P-1, which will start operation in 2026–2027 observational season. The effective area and angular resolution of P-1 are shown as a function of γ-ray energy in (B) and (C), respectively. Three colors represent cases at zenith angles of 50°, 60°, and 70°, respectively.

The LACT experiment, consisting of 32 IACTs, is being deployed on the LHAASO site at 4,410 m above sea level as depicted in Fig. 3A. The 6-m-diameter reflector of each telescope is formed with 54 facet mirrors with the Davies–Cotton design. Each facet is a hexagonal spherical mirror with the curvature radius of 16 m and a side length of 0.46 m. The telescope camera has 1,616 pixels made of silicon photo multipliers. Each pixel covers $0.19^{\circ }\times 0.19^{\circ }$, thus forming the field of view (FOV) of 8° in diameter for each telescope. For the detailed description of LACT, please see [14].

The telescopes of LACT are deployed in 8 groups of 4 telescopes forming a quasi-square grid with a distance of ~140 m between 2 adjacent telescopes. The average distance between 2 neighboring group is about 420 m. At the altitude of 4,410 m, vertical showers are characterized by their steep lateral distribution of Cherenkov photons. Therefore, every group individually measures showers, with at least 3 telescopes in a group detecting photons from the same shower, which has the threshold energy around 0.1 TeV for γ rays.

Showers arriving at large zenith angles typically possess higher energies and cover a broader area. As a result, the lateral distribution of Cherenkov light becomes flatter, allowing for a widely spaced telescope array. This configuration maximizes the effective detection area of the whole array. Above ~100 TeV, the effective collection area can be as large as 5 km^2^; thus, the best performance of the array is achieved in the UHE regime. As shown in Fig. 3A, 32 telescopes are grouped into 4 nearly symmetric sub-arrays (labeled by different colors) with larger distance between telescopes, typically 420 m in average. Each sub-array operates independently by tracing different sources simultaneously.

The phase-1 (P-1) of LACT is under construction now, containing 4 telescopes (marked by cyan circles in Fig. 3A), and will be completed in 2026. Very good performance for observations of showers coming with large zenith angle is expected as illustrated in Fig. 3B and C. The collection area can achieve 3 km^2^ at 100 TeV for a zenith angle of 60° or larger. On top of this, the angular resolution reaches about 0.1° at such high energies.

## Data Availability

All data necessary to evaluate the conclusions of this study are available within the article or its referenced literature. There are no restrictions on data availability.
